# Hierarchical Ag Nanostructures Fabricated from Silver Coordination Polymers for Antibacterial Surface

**DOI:** 10.3390/polym11010155

**Published:** 2019-01-17

**Authors:** Jin-Song Jung, Su-Joung Ko, Hong-Beom Lee, Su-Bin Lee, Hyoung-Jun Kim, Jae-Min Oh

**Affiliations:** Department of Chemistry and Medical Chemistry, College of Science and Technology, Yonsei University, Wonju, Gangwondo 26493, Korea; jjs406@yonsei.ac.kr (J.-S.J.); alsbalxl@naver.com (S.-J.K.); stb91009@gmail.com (H.-B.L.); sban0103@naver.com (S.-B.L.)

**Keywords:** silver nanoparticle, silver coordination polymer, reductive calcination, hierarchical structure, antibacterial activity, surface coating

## Abstract

A hierarchical silver nanostructure with improved antibacterial property was fabricated utilizing silver coordination polymer. Octadecanethiolate–silver polymer was synthesized to have a layered structure and was coated on silicon wafer by drop-casting method utilizing hydrophobic–hydrophobic interaction. Thus, the silver coordination polymer was calcined under reductive condition to produce zero-valent silver with a hierarchical nanostructure. X-ray diffraction patterns revealed that layered silver coordination polymer successfully transformed to hexagonal silver upon calcination. According to scanning electron and atomic force microscopy, silver coordination polymer with ~145.5 nm size was homogeneously coated on the surface before calcination, and it evolved micrometer-sized lumps and grooves which were composed of ~58.8 nm sized Ag nanoparticles. The hierarchical structure—micrometer lump/groove consisting of Ag nanoparticles—would be advantageous to kill bacteria; micrometer-grooves provide physical condition (pocket for bacteria capture) and the Ag nanoparticles from the neighboring lump endow chemical condition (antibacterial property of released Ag^+^). The antibacterial activity test on *Escherichia coli* via colony forming inhibitory assay indeed exhibited an improved antibacterial activity of hierarchical Ag nanostructure compared with the surface simply coated with Ag nanoparticles. From the line profile of atomic force microscopy, the bacterium trapped in the hierarchical Ag nanostructure was shown to interact intimately with Ag surface.

## 1. Introduction

Silver nanoparticle (AgNP) is one of the widely utilized nanomaterials having antibacterial, catalytic, optical properties, and so on [1,2,3]. Among them, antibacterial property of AgNPs has long been applied to commercial products such as fabrics, cosmetic materials, toothpastes, filters, kitchen utensils, shampoos, and humidifiers [4,5,6,7]. Antibacterial mechanism of AgNPs is known to derive from dissolved silver ion; monovalent silver ion is dissolved from the surface of AgNPs having large specific surface area, and the ion can kill microbes in various ways. Some researchers support the idea that the released silver cations interact with the thiol groups in bacteria proteins, affecting the replication of DNA [8]. It has also been reported that silver ions were bound to the bacterial cell wall and outer bacterial cell, changing the function of the bacterial cell membrane [9,10,11]. In any case, AgNPs surely contain antibacterial property in the material point of view.

In order to provide consumer product with AgNPs’ antibacterial property, several strategies could be applied. Ashavani and co-workers reported an ecofriendly chemical approach to synthesize AgNPs embedded paint from common household paint. The naturally occurring oxidative drying process in oils, which involves free-radical exchange, is the fundamental mechanism for reducing silver salts and dispersing AgNPs in the oil media, without the use of any external reducing or stabilizing agents [12]. Bacterial cellulose is a natural hydrogel whose properties are better than those produced from synthetic polymers. However, bacterial cellulose itself has no antimicrobial activity to prevent wound infection. To achieve an antimicrobial activity, silver nanoparticles were impregnated into bacterial cellulose through the chemical reduction; bacterial cellulose immersed in silver nitrate solution reduced Ag^+^ to Ag^0^ for AgNP production [13]. Silver nanoparticles can be coated on commercial polyurethane (PU) foams by overnight exposure of the foams to nanoparticle solution. Repeated washing and air-drying yielded PU foam in which AgNPs were uniformly coated [14]. The AgNPs were reported fairly stable on the PU foam and were not easily washed away by water. Authors suggested that the material can be used as a drinking water filter, where bacterial contamination of the surface water is a health risk.

Although there have been many approaches to introduce AgNPs in various materials, these approaches stay in the exploitation of AgNPs’ chemical-antibacterial property. In addition to those approaches, physical manipulation of surface property has been suggested to improve functionalities. A recently proposed method in antibacterial or antifouling is surface patterning, which states that the performance is dependent on the surface of micro/nanostructure within the same material. An example is to establish a hierarchical structure. In this regard, hybridizations between hierarchical materials and various antibacterial metals have been reported. Hongwei and co-workers demonstrated hierarchical corn-like material in which Cu nanoparticles were grown in situ on the ZnO nanorod substrate [15]. This material exhibited not only maximized photocatalytic activity but also antibacterial activity compared with Cu nanoparticle only or ZnO nanorod itself. In another research, Ferdi et al. reported that hierarchical three-dimensional nanopillared surface on titanium made a greater exposure of the antibiotic coating to adhered bacteria, which led to an increase in antibacterial efficiency [16]. On this wise, design of hierarchical structure with antibacterial metal nanoparticle is considered the optimal way to obtain antibacterial property. To our knowledge, however, hierarchical structure with silver nanoparticles has not been reported systematically.

Silver–alkanethiolate is fairly characteristic as its two-dimensional structure could influence the morphology of silver nanostructures after thermal reduction. For instance, Wu and co-workers prepared thin silver nanodisks via thermal treatment of silver–alkanethiolate with dodecane chain at 180 °C under N_2_ atmosphere [17]. Likewise, silver–alkylthiolate is expected to be a precursor for various Ag nanostructures. In the present paper, we especially chose silver–octadecanethiol, as long alkyl chain is considered to stabilize layered structure through van der Waals interaction. We expected that hierarchical silver structure would be effectively obtained deriving from the stabilized structure of silver–octadecanethiolate polymer. AgNP was combined with hierarchical structure to obtain improved antibacterial property. To achieve this purpose, AgNP was synthesized starting from silver coordination polymer [18,19], as silver–alkylthiolate polymer is reported to be transformed to fibrous hierarchical structure upon reductive calcination. As the silver coordination precursor, octadecanethiolate–silver coordination polymer (AgSC18) was prepared and coated on a certain surface. The AgSC18 moiety on the surface was calcined under reductive gas condition to obtain hierarchical nanostructure of Ag. Through X-ray diffractometry and microscopies, we traced the structure, morphology, and surface topology of coated surface. For comparison, we also prepared surface coated with Ag nanoparticles. Both surfaces were subjected to antibacterial colony forming inhibition assay and atomic force microscopy analyses after bacteria *Escherichia coli* exposure. The correlation between antibacterial property and surface hierarchical structure will be discussed in detail.

## 2. Materials and Methods

### 2.1. Preparation of Silver Coordination Polymer

Alkylthiolate–silver coordination polymer was prepared referring to the previous literatures [19]. Silver nitrate (AgNO_3_, 4.9 × 10^−3^ mol; Sigma-Aldrich, Inc., St. Louis, MO, USA) was dissolved in a mixed solvent (50 mL of tetrahydrofuran (THF; Daejung Chemicals & Metals CO., LTD., Siheung, South Korea) and 25 mL of ethanol (EtOH; Daejung Chemicals & Metals CO., LTD., Tokyo South Korea)), and octadecanethiol (9.1 × 10^−3^ mol; Sigma-Aldrich, Inc., St. Louis, MO, USA) was dissolved in 50 mL of THF solvent. Both solutions were mixed and vigorously stirred under ambient condition. After 24 h, the obtained precipitate was separated by centrifugation and unreacted octadecanethiol moiety was washed out utilizing THF and EtOH. Thus obtained product, AgS(CH_2_)_17_CH_3_ coordination polymer (AgSC18) was dried in vacuum oven.

### 2.2. Surface Coating with Silver Nanostructures

Silver coordination polymer was coated on the silicon wafer surface. For effective introduction, silicon wafer (Si; Silicon Technology CO., LTD., Tokyo, Japan) was cleaned with piranha solution (H_2_SO_4_ (Sulfuric acid; Daejung Chemicals & Metals CO., LTD., Siheung, South Korea): H_2_O_2_ (Hydrogen peroxide; Samchun Chemicals CO., LTD., Seoul, South Korea) = 3:1) and silanized with octadecanetriethoxysilane (Tokyo Chemical Industry CO., LTD., Tokyo, Japan) in toluene (Sigma-Aldrich, Inc., St. Louis, MO, USA) solvent. The reaction was carried out under dehydrated condition at 115 ℃ for 12 h.

For coating process, 20 μL of AgSC18/THF suspension (0.033 g/mL) was dropped on the silicon wafer (1 cm × 1 cm) and spread utilizing 20-μm-thick applicator. Solvent was dried under ambient condition for 30 min (AgSC18@Si). The specimen was thermally treated at 500 ℃ for 4 h under reductive gas condition (N_2_:H_2_ = 9:1). Thus, the obtained specimen is designated Ag-hc@Si considering the expected hierarchcial (hc) silver structure. For the comparison, silver nanopowder (AgNP; ~50 nm size, Kojima Chemicals CO., LTD., Japan) was suspended in THF with equimolar silver concentration compared with AgSC18. The suspension was applied to the cleaned silicon wafer and dried at room temperature for 30 min (AgNP@Si).

### 2.3. Characterization

In order to confirm the phase and crystallinity of both samples in powder and film state, X-ray diffractometer (XRD; D2 phase, Bruker, Germany) was utilized with 1 mm air-scattering slit and a 0.1 mm equatorial slit. XRD patterns were obtained from 3° to 80° (2θ) with time step 0.5 s/°.

Functional groups of AgSC18 were investigated by Fourier transform infrared spectroscopy (FTIR; Spectrum 65 FTIR Spectrometer, PerkinElmer, Waltham, MA, USA). The scanning range was 400–4000 cm^−1^ using a spectral resolution of 8 cm^−1^. In total, 64 scans were averaged for each FTIR spectrum.

Particle size, morphology, and distribution of samples were investigated with scanning electron microscopy (SEM; FEI Quanta 250 FEG, Hillsboro, OR, USA). Energy-dispersive spectrometer (EDS; Apollo X, AMETEK, PA, USA) was utilized to figure out the chemical composition. Powdered sample was suspended in EtOH and dropped on pre-cleaned silicon wafer. Film samples were measured after Pt/Pd plasma sputtering for 60 s.

Surface topology of film samples were examined by atomic force microscopy (AFM; NX10, Park systems, South Korea). The film samples were measured without further treatment, and the AFM was managed under non-contact mode.

### 2.4. Evaluation of Antibacterial Activity of Ag Nanostructure Coated Surface

The antibacterial activity was evaluated with Gram-negative *E. coli* bacteria utilizing colony forming inhibitory assay. Each specimen (1 cm × 1 cm) was located in a 6-well plate (well diameter, 3.5 cm) with the coating layer upside, and then sterilized with ultraviolet irradiation for 30 min. Suspensions of *E. coli* (3.3 × 10^5^ bacteria/mL) were inoculated in Luria-Bertani (LB) broth culture media and incubated at 37 °C for 6 h. The well without specimen was utilized as a control group. Then, the incubated bacteria suspension was collected, diluted to 1.0 × 10^3^ bacteria/mL, and spread on culture agar plates. The agar plates containing bacteria were incubated at 37 °C for 12 h. The number of colonies was counted utilizing a digital camera and a colony counting program (Image Processing and Analysis in Java; ImageJ) to calculate the antibacterial activity. All the antibacterial tests were triplicated. To confirm the difference in antibacterial activity among samples, Student’s t-test with confidence level 99.9% was applied.

In order to visualize topology and location of *E. coli* on each surface, AFM images were obtained. Each sample located in a 6-well plate was submerged beneath the suspension of *E. coli* (3.3 × 10^5^ bacteria/mL) and incubated at 37 °C for 6 h. After each sample was relocated in a new 6-well plate, it was treated with 4% paraformaldehyde on its surface and kept in the refrigerator for 20 min for fixation. The bacteria-fixed samples were dried under room temperature and subjected to AFM measurement under non-contact mode.

The biological resources used in this research were distributed from KCTC (*E. coli*, resource number 2571).

## 3. Results and Discussion

### 3.1. Hierarchical Ag Structure from Ag Coordination Polymer

**Figure 1:**
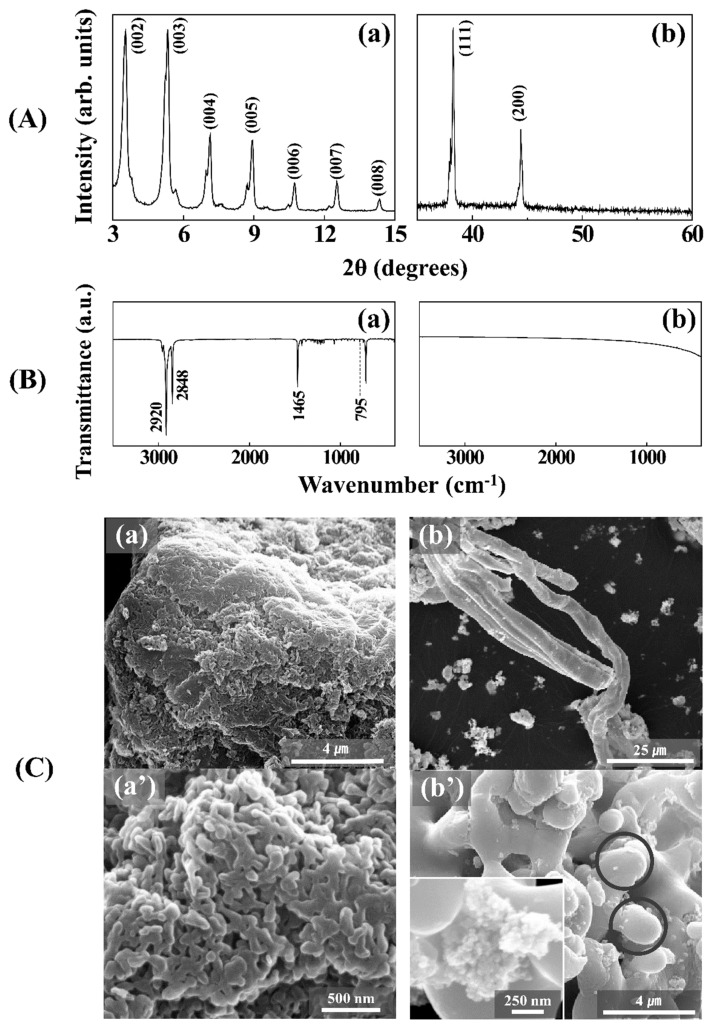
(**A**) X-ray diffraction patterns, (**B**) Fourier transform infrared spectra, and (**C**) scanning electron microscopic images of (**a**) AgSC18 and (**b**) calcined AgSC18 in powder state. (**C**(**a’**,**b’**)) are magnified images corresponding to (**a**) and (**b**). For the calcination, powder state AgSC18 was thermally treated at 500 °C for 4 h under reductive gas condition (N_2_:H_2_ = 9:1). The solid circle and inset in (**C**(**b’**)) indicate micrometer- and nanometer-sized Ag particles, respectively.

Structure and morphology of Ag obtained from Ag coordination polymer in powder state was confirmed by XRD and SEM study (Figure 1). As shown in Figure 1A(a), as prepared AgSC18 showed well-developed (00l) peaks due to the layered structure as reported with other silver–alkylthiolate coordination polymer [20,21]. The d-spacing of (001) was calculated 49.9 Å, which was corresponding to the layer thickness (~4.5 Å) and chain length of octadecyl (~28 Å) with a tilting angle ~54°. We could confirm that the layered structure of AgSC18 was maintained through van der Waals interaction among alkyl moiety.

FTIR and EDS were measured in order to investigate the functional groups and chemical composition of AgSC18. FTIR spectra of coordination polymer (Figure 1B(a)) clearly showed IR absorption at 2920, 2848, 1465, and 795 cm^−1^ attributed, respectively, to –CH_3_ stretching (for both 2920 and 2848 cm^−1^), –CH_2_– wagging, and C–S bending vibrations. The EDS analysis result indicated that the coordination polymer has 8.00% and 57.2% of S and C, respectively, which were almost similar to the theoretical value of 8.15% and 55.0%.

The SEM images for AgSC18 (Figure 1C(a,a’)) revealed that the particles were fairly small (~145.5 nm) with irregular shapes. After calcination of AgSC18 under reductive gas (N_2_:H_2_ = 9:1), we could obtain zero-valent Ag with a characteristic morphology. As reported previously [19,22,23], gaseous reduction of alkylthiolate–silver coordination polymer resulted in the growth of fibrous Ag structure. The XRD (Figure 1A(b)) showed characteristic (111) and (200) peaks corresponding to face-centered cubic structure of Ag with lattice parameter, a = 4.1 Å. The SEM images exhibit bundle fibers (Figure 1C(b)) of which terminal (Figure 1C(b’)) was composed of both micrometer- (solid circle) and nanometer-sized particles (inset). Fiber is an assembly of micrometer-sized particles, which are composed of nanometer-sized particles; thus, the silver obtained from alkylthiolate–silver coordination polymer is thought to form a hierarchical nanostructure.

### 3.2. Hierarchical Ag Structure on Surface Starting from Coordination Polymer

**Scheme 1:**
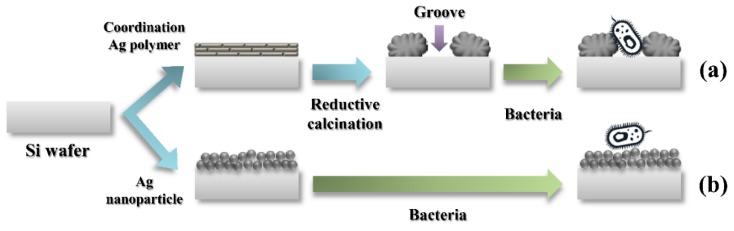
Schematic diagram to obtain a surface with hierarchical silver nanostructure or silver nanoparticles. (**a**) AgSC18 coordination polymer was coated on the surface and zero-valent silver was obtained by reductive calcination; the obtained hierarchical structure with appropriate groove can capture bacteria and kill them with the chemical action of silver. (**b**) Silver nanoparticle was directly applied to the surface; the nanoparticles were randomly distributed on the surface, and thus there was less contact between silver and bacteria resulting in limited antibacterial effect.

As we confirmed that the reductive calcination of silver coordination polymer gave rise to hierarchical structure which was effective in antibacterial property, we applied the process to surface coating. Scheme 1 briefly summarized the coating process utilizing either silver coordination polymer or silver nanoparticles. Silver coordination polymer (AgSC18), due to its layered structure, would be coated flat on the surface. In powder state, reduction of AgSC18 occurred along with the crystal growth in a certain direction. However, the surface coated AgSC18 would be gathered at certain points resulting in pebble-like silver particles. Thus, the obtained pebble-like lumps would be composed of Ag nanoparticles, and the distribution of lumps constructed the inter-particle groove, the so-called hierarchical nanostructure. If the obtained groove was appropriate to the dimension of bacterium, the contact area between bacterium and silver would be maximized (Scheme 1a). Supposing that the silver nanoparticles were directly applied on the surface, there would be a homogeneous distribution of AgNP, resulting in the limitation of contact area between silver and bacteria (Scheme 1b).

**Figure 2:**
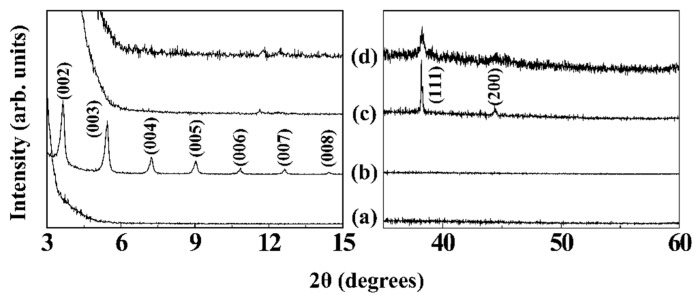
X-ray diffraction patterns of (**a**) bare Si wafer, (**b**) AgSC18@Si, (**c**) Ag-hc@Si, and (**d**) AgNP@Si. 2θ range between 15° and 35°, where intense silicon single crystal peaks from substrate wafer were shown, was intentionally omitted.

In order to confirm the crystal phase and crystallinity of samples on the surface, we traced the XRD patterns. First of all, the silicon wafer (Si) itself did not show any significant diffraction pattern in the 2θ range measured, 3~15° and 35~60° (Figure 2a). Although, there were strong peaks at ~34.5° and ~69.0° corresponding to (200) and (400) of Si, respectively, we excluded the range to clearly show the diffraction patterns of samples, silver coordination polymer, and zero-valent silver. The XRD pattern of AgSC18@Si was almost same as that of powder state AgSC18 (Figure 1A(a)), showing repeated (00l) peaks and d-spacing corresponding to 48.5 Å (Figure 2b). It was noteworthy that there was no significant diffraction at a higher angle region (35~60°). This is attributed to the orientation of AgSC18 particles through crystallographic a-axis due to their layered structure and inter-particle van der Waals interaction at the surface. Upon reductive calcination (Figure 2c), there disappeared (00l) peaks from silver coordination polymer and newly appeared peaks corresponding to (111) and (200) of hexagonal Ag structure at 38.2° and 44.4°, respectively. Silver nanoparticle coated surface also showed hexagonal Ag peaks, (111) and (200). The crystallite sizes along (111) direction utilizing Scherrer’s equation (t = (0.9 × λ)/(Bcosθ), where t, crystallite size; λ, X-ray wavelength; B, full-width at half-maximum of peak; θ, Bragg angle) were 61.6 and 25.3 nm, respectively, for Ag-hc@Si and AgNP@Si.

**Figure 3:**
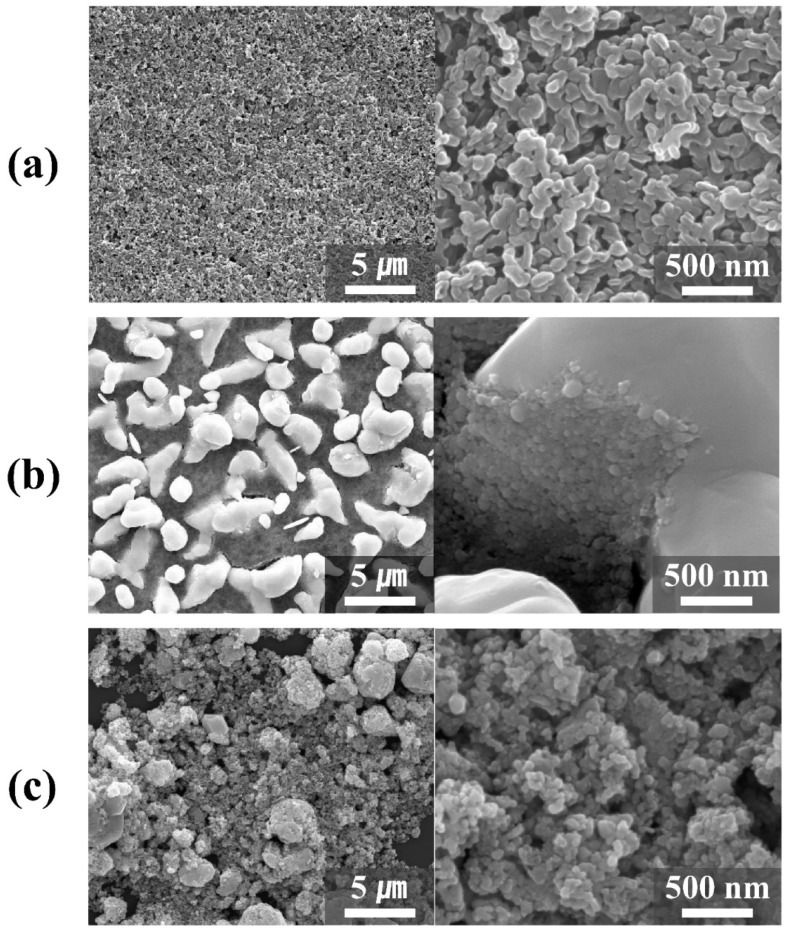
Scanning electron microscope images for surface of (**a**) AgSC18@Si, (**b**) Ag-hc@Si, and (**c**) AgNP@Si.

Surface morphology of each coated sample was investigated by SEM measurement. The silicon wafer coated with AgSC18 showed a homogeneous distribution of particles with an average size of 145.5 nm (Figure 3a). There was no significant change in particle size and morphology upon drop casting process. After reductive calcination (Ag-hc@Si), we could observe large particles with a few micrometers dimension (Figure 3b, left panel). The particles were randomly distributed like scattered pebbles. If the AgSC18 was calcined in powder state, they would grow along a certain direction showing a fibrous morphology. However, the surface attached AgSC18 would not have enough chance to get together, and thus they would show a regional crystal growth. Thanks to this phenomenon, there were micrometer-sized grooves among big lumps, and the dimension of grooves corresponded to the size of bacteria. It was worthy to note that the micrometer-sized particle was formed by the assembly of nanoparticles as shown in the magnified image (Figure 3b, right panel). The order of dimension (few tens of nanometers) corresponded to the crystallite size calculated by Scherrer’s equation from XRD data. Due to this hierarchical structure of Ag nanoparticles and large lumps, it was expected that the captured bacteria in the groove would encounter large surface of Ag nanoparticles, resulting in growth inhibition. Figure 3c shows the surface of AgNP coated silicon wafer. We could clearly observe that the aggregates of AgNP were randomly distributed throughout the surface. There was neither regular nor hierarchical structure of Ag particles. In this regard, the bacteria attached on the surface of AgNP@Si would confront less chance of Ag’s antibacterial effect.

**Figure 4:**
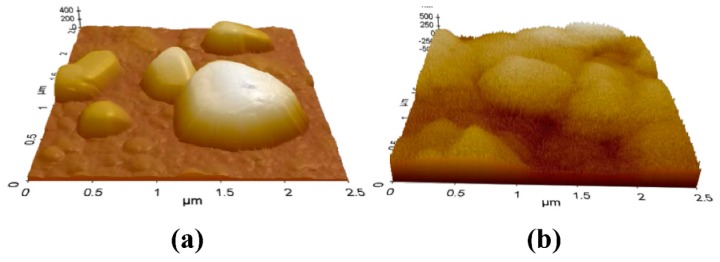
Three-dimensional atomic force microscopic (AFM) topography of (**a**) Ag-hc@Si and (**b**) AgNP@Si.

Although it was clearly shown that the surface morphology of Ag-hc@Si and AgNP@Si were different from each other as shown in Figure 4, we further examined surface topology with AFM in three-dimensional image mode. The surface of Ag-hc@Si clearly showed that there exist both large lumps and small particles on the surface of groove; on the other hand, AgNP@Si showed aggregates of particles which did not show a typical topology.

### 3.3. Antibacterial Effect of Hierarchical Silver Structure

**Figure 5:**
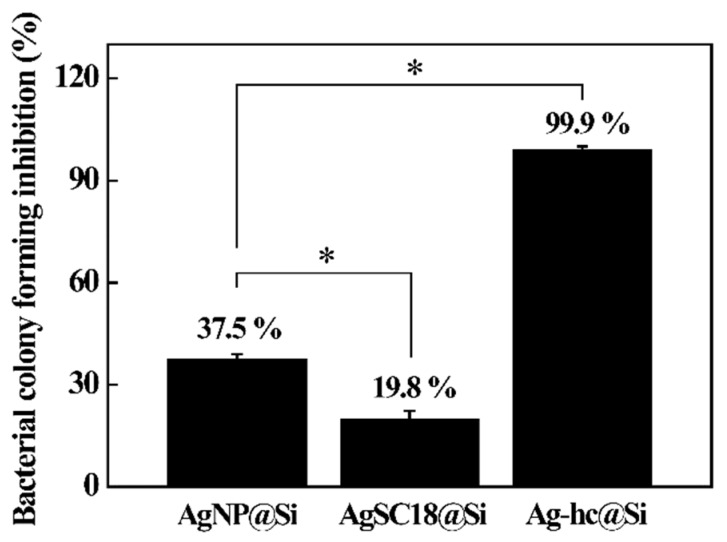
Bacterial colony forming inhibition (%) of AgNP@Si, AgSC18@Si, and Ag-hc@Si for *Escherichia coli*. Each specimen was immersed into *E. coli* suspension for 6 h. Asterisk stands for statistical difference in 99.9% of confidence interval.

To compare the antibacterial activities of silver coated surface, we carried out bacterial colony forming inhibition assay on *E. coli*. The Student’s t-test was utilized for statistical comparison. Figure 5 exhibits that Ag-hc@Si has the highest antibacterial efficacy among the tested samples showing 99.9% of value. Both AgNP@Si and AgSC18@Si revealed less than 40% of bacterial inhibition. AgSC18@Si showed the lowest colony forming inhibition, which was attributed to the Ag^+^ ions tightly bound to the coordination polymer. Slight inhibition effect would be due to the alkyl chain at the surface which could attack the cell membrane. AgNP@Si showed a better antibacterial effect (in confidence level 99.9%) than AgSC18@Si due to the Ag^+^ release from AgNP; however, the efficacy is fairly low as there was less chance for Ag^+^ ion to meet bacteria. It should be noted that the Ag-hc@Si showed clearly distinguished antibacterial activity in spite of the equivalent amount of Ag in each sample (0.18 mg Ag/cm^2^ surface for all the three samples). This result strongly suggested that both the physical effect (hierarchical structure) and the chemical effect (Ag+ release from nanoparticle) played important roles in the antibacterial effect. The size of large silver lump was as large as 4 μm and the groove among lumps was appropriate for the micrometer-sized bacterium, i.e., *E. coli* to be captured. Each lump of silver was composed of nanosized silver particles. Once trapped, *E. coli* would encounter Ag^+^ cation released from every side of Ag particles (from the wall of lumps and the surface of groove) to be strongly suppressed of its proliferation.

**Figure 6:**
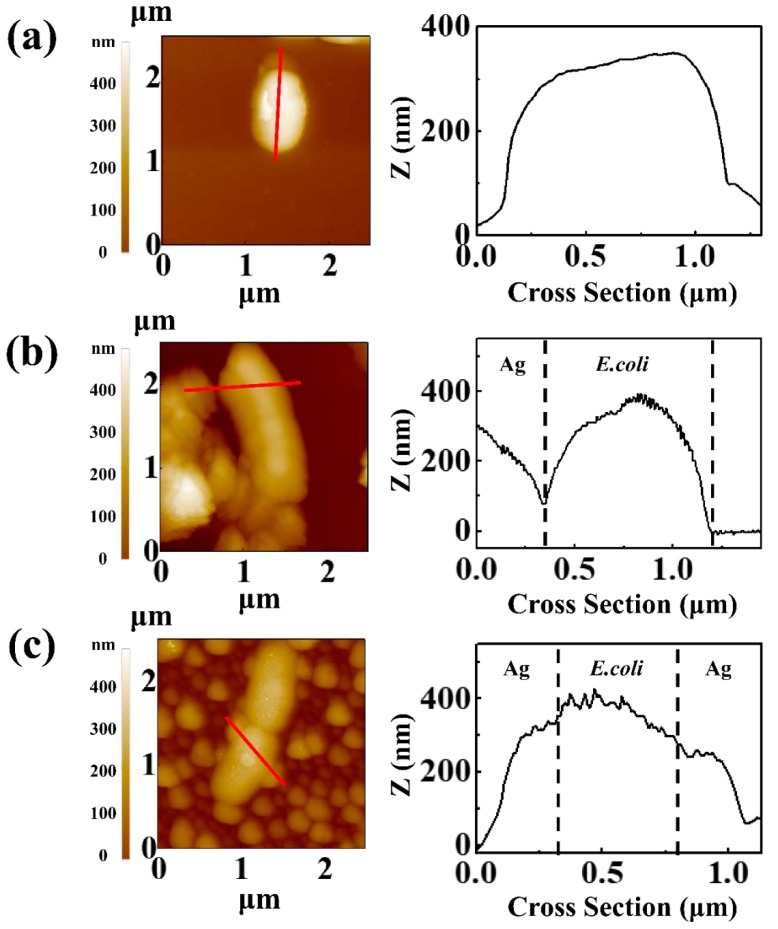
AFM topography (**left**) and line profile along red line (**right**) for *E. coli* on (**a**) bare Si, (**b**) AgNP@Si, and (**c**) Ag-hc@Si.

Tight junction between silver structure and *E. coli* was visualized by AFM (Figure 6). The size of *E. coli* was around 1 μm in length and 400 nm in thickness (Figure 6a). The surface of bare *E. coli* was fairly smooth (line profile of Figure 6a). Once they contacted the silver nanoparticle, the surfaces became rough (line profiles of Figure 6b,c), which is the signal of cell death [24,25]. It was interesting that the *E. coli* on Ag-hc@Si was captured among Ag lumps, and there was a tight junction between Ag and *E. coli* (Figure 6c). On the other hand, the junction between Ag and *E. coli* was less interactive in AgNP@Si (Figure 6b). Thus, we could conclude that the hierarchical structure enabled more active interaction between bacteria and surface Ag nanoparticles.

Damm and co-workers reported about polyamide 6 in which silver-nano- and microcomposites were used as fillers [26]. They claimed that silver microparticles showed only ~40% of antibacterial property, while nanoparticles showed almost 100% of activity. In our research, silver microparticles were obtained on the surface; however, due to the hierarchical structure of nanoparticles, the surface showed 99.9% of antibacterial activity. The result was corresponding to that of Damm et al., but our result was more fascinating because silver microparticles with hierarchical nanostructures showed a strong antibacterial property.

## 4. Conclusions

In this work, we have successfully prepared hierarchical surface (Ag-hc@Si) via reductive calcination of silver coordination polymer. While silver nanoparticle coated surface (AgNP@Si) showed random distribution of particles with uncontrollable aggregates, surface of Ag-hc@Si clearly revealed that there existed hierarchical structure of large lump/groove composed of Ag nanoparticles. As the dimensions of lumps and grooves were suitable for the micrometer-sized bacteria, *E. coli*, the bacterial cells could be effectively trapped in the groove site, which was visualized with the AFM line profile. As a matter of course, the silver nanoparticle with hierarchical structure (Ag-hc@Si) showed much improved antibacterial activity of 99.9% than the surface coated with equivalent amount of silver (AgNP@Si) having antibacterial activity ~37.5%. In conclusion, we successfully fabricated hierarchical silver nanostructure starting from silver coordination polymer, and the antibacterial efficacy was improved by its unique morphology. (See Supplementary Materials).

## Supplementary Materials

The following are available at http://www.mdpi.com/2073-4360/11/1/155/s1.
