# Supplementary material for: Hierarchical Ag Nanostructures Fabricated from Silver Coordination Polymers for Antibacterial Surface
Source: Polymers (Basel). 2019 Jan 17;11(1):155. doi: 10.3390/polym11010155 (PMC6401719; doi:10.3390/polym11010155)
Supplement: Supplementary file 1 [file polymers-11-00155-s001.pdf]

Microanalysis Report

Prepared for: ***Company Name Here***

Prepared by: ***Your Name Here*** ***1/10/2019***

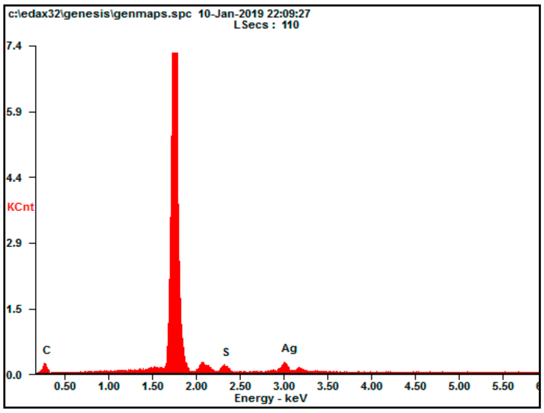

Element	*Wt%*	*At%*
CK	57.16	89.26
SK	08.00	04.68
AgL	34.84	06.06
Matrix	Correction	ZAF

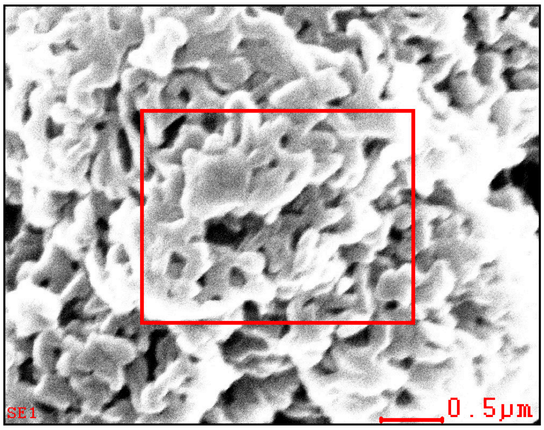

Chemical formula for Ag-C18:$C_{18}H_{37}S$·Ag

Element	Theoretical value (wt%)	EDS analysis (wt%)
**C**	**55.0**	**57.2**
**S**	**8.15**	**8.00**
**Ag**	**27.4**	**34.8**
